# Staged surgical repair of severe pulmonary stenosis post radiofrequency ablation

**DOI:** 10.1093/jscr/rjac390

**Published:** 2022-09-05

**Authors:** Lewis William Murray, Rakesh Gopal, Damian Gimpel, Malgorzata (Maggie) Szpytma, Gareth Crouch

**Affiliations:** Department of Cardiothoracic Surgery, Flinders Medical Centre, Adelaide, SA, Australia; Department of Cardiothoracic Surgery, Flinders Medical Centre, Adelaide, SA, Australia; Department of Cardiothoracic Surgery, Flinders Medical Centre, Adelaide, SA, Australia; Department of Cardiothoracic Surgery, Flinders Medical Centre, Adelaide, SA, Australia; Department of Cardiothoracic Surgery, Flinders Medical Centre, Adelaide, SA, Australia

## Abstract

We present a case of a 68-year-old man who presents with worsening cough and dyspnoea 12 months after undergoing radiofrequency ablation therapy for atrial fibrillation. Investigation revealed complete occlusion of the left lower pulmonary vein and partial stenosis of the left upper pulmonary vein. He underwent a stage surgical resection with the first stage being a left lower lobectomy for the non-viable lobe followed by a repair of the left upper pulmonary vein via anastomosis with the left atrial appendage. This staged procedure yielded excellent results and avoided the need for a left-sided pneumonectomy.

## INTRODUCTION

The formation of pulmonary vein stenosis following radiofrequency ablation (RFA) therapy for patients with atrial fibrillation (AF) is a recognized complication. The mechanism of stenosis formation can occur in either the early or late post procedural phase. It is driven by myocardial necrosis and this acute inflammatory process can promote thrombus formation in the early stage. Late-stage stenosis results from the fibrotic changes associated with myocardial fibrosis that can also involve the pulmonary veins which leads to venous contraction and stenosis.

We present a case of staged repair of a left upper and lower pulmonary vein stenosis which formed secondary to percutaneous RFA which preserved the functional left upper lobe and avoided the need for a left pneumonectomy.

## CASE REPORT

A 68-year-old man with a history of refractory paroxysmal AF and severe left ventricular dysfunction underwent catheter-guided percutaneous RFA. After initial improvement in symptoms and left ventricular (LV) function, over a period of 12 months the patient developed persistent cough and worsening exertional dyspnoea presenting with NYHA III symptoms.

Repeat transthoracic echocardiogram demonstrated no deterioration in left ventricular function to coincide with symptom progression. A computed tomography (CT) chest demonstrated patchy infiltrates in the left lower lobe and a small pleural effusion. After multi-disciplinary team (MDT) presentation, a dedicated CT pulmonary vein study was obtained demonstrating pulmonary vein stenosis of the upper left pulmonary vein and complete occlusion of the lower left vein. A ventilation perfusion (V/Q) scan confirmed the lower lobe to be non-functional but the upper lobe still viable. A staged procedure was planned to avoid the need for a pneumonectomy.

**Figure 1:**
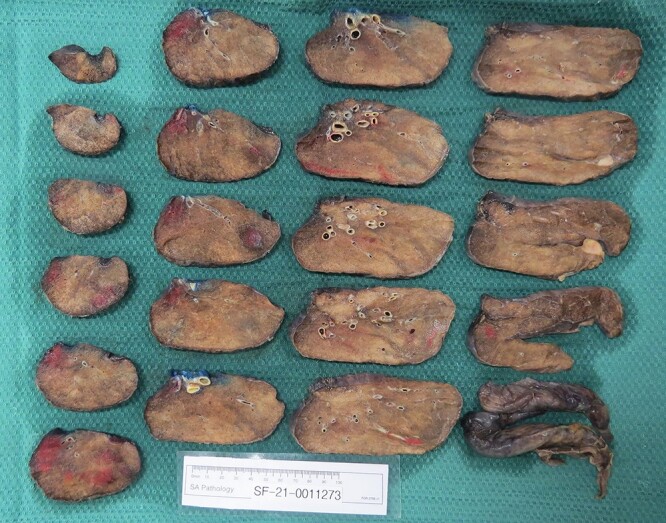
Histological assessment of left lower lobe.

The patient underwent a staged left lower lobectomy and pulmonary vein repair as a staged procedure. The left lower lobe was resected as a video-assisted thoracoscopic procedure. Intraoperatively, the left lower lobe was oedematous with dense inflammatory adhesions to the chest wall. During the lobectomy, the left upper pulmonary vein was dissected in preparation for the planned upper pulmonary vein repair. Histopathology confirmed the left lower lobe to be necrotic (Fig. 1).

**Figure 2:**
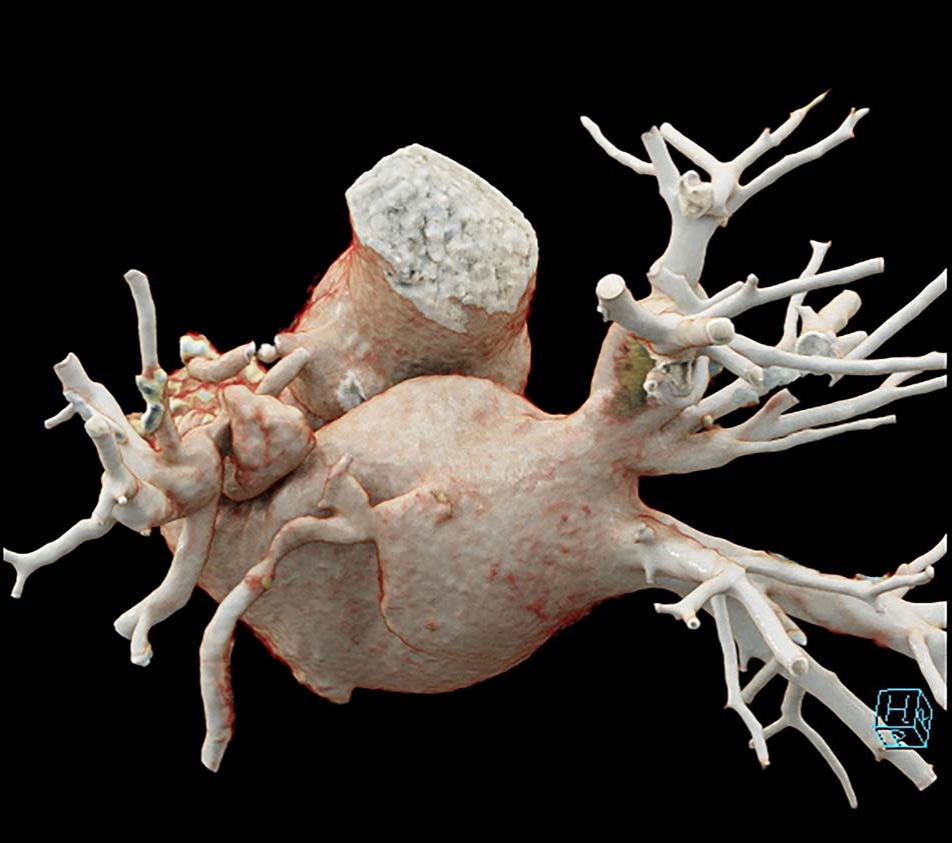
3D reconstruction of anastomosis of pulmonary vein to left atrial appendage.

The patient was returned to the operating room 3 days later to undertake repair of the left upper pulmonary vein stenosis via sternotomy. The patient was placed onto cardiopulmonary bypass using antegrade Del Nido cardioplegia. Opening of the left atria revealed a circumferential stenosis of the left upper pulmonary vein. The vein was stapled at the left atrial junction and then trimmed off. The left atrial appendage was opened, several minor trabeculations were resected and the left upper pulmonary vein anastomosed to the appendage (Fig. 2). When weaned from bypass, the gradient across the anastomosis was measured at 1 mmHg. The patient recovered without complication.

## DISCUSSION

Catheter RFA is an effective treatment modality for both permanent and paroxysmal AF in patients who have been unsuccessfully treated with antiarrhythmic drug therapy [1]. Randomized trials have gone on to demonstrate catheter RFA is also effective as a first-line treatment [2]. Catheter RFA has been demonstrated to be an effective treatment for AF but can be complicated by pulmonary vein (PV) stenosis with rates ranging from 1% to 21% as reported in the literature [3]. Alternatively, the use of cryoablative therapy should theoretically have a lower incidence of pulmonary vein stenosis due to the lack of muscular contraction during the healing process; however, cases have still been reported [4]. The wide variation of incidence is due to the lack of clear guidelines for post ablation screening. The symptomatology of PV stenosis is also wide ranging and often vague, adding to the difficulty of diagnosis, especially in patients with co-existing air-way disease [5].

PV stenosis can be managed with balloon dilation or venous stenting. Both procedures carry a risk of restenosis with rates of >50% balloon dilation and > 25% reported [6]. Early intervention is recommended to restore pulmonary blood but for late presentations with stenosis of >95% then lobectomy is indicated [7]. This case demonstrates that adverse outcomes can be associated with RFA. Importantly, this case demonstrates a unique staged surgical approach to the treatment of severe pulmonary vein stenosis post RFA allowed for the preservation of the viable upper lobe and thus avoiding the morbidity due to reduced lung capacity, a pneumonectomy as well as direct treatment of the stenosis by anastomosing the pulmonary vein to the left atrial appendage.
